# Real-Time Comparison of Machine Learning-Enabled Devices for Measuring Compensatory Reserve Status

**DOI:** 10.3390/bioengineering13070817

**Published:** 2026-07-16

**Authors:** Carlos Bedolla, Jose M. Gonzalez, Ryan Ortiz, Krysta Amezcua, Sofia I. Hernandez Torres, Victor A. Convertino, Eric J. Snider

**Affiliations:** 1 Expeditionary Medical Systems Department, U.S. Army Institute of Surgical Research, JBSA Fort Sam Houston, San Antonio, TX 78234, USA; 2 Battlefield Health & Trauma Center for Human Integrative Physiology, U.S. Army Institute of Surgical Research, JBSA Fort Sam Houston, San Antonio, TX 78234, USA; 3 Department of Medicine, Uniformed Services University of the Health Sciences, Bethesda, MD 20814, USA; 4 Department of Emergency Medicine, University of Texas Health Science Center, San Antonio, TX 78229, USA; 5 Department of Surgery, University of Texas Health Science Center, San Antonio, TX 78229, USA

**Keywords:** central hypovolemia, noninvasive sensing, triage algorithm, decompensation, hemorrhagic shock, medical device testing

## Abstract

In this work, the real-time measurement of compensatory reserve metrics is compared during a simulated central hypovolemia clinical research study. We hypothesized that values generated from the compensatory reserve measurement (CRM) device would be statistically similar to those values generated from the compensatory reserve index (CRI) device. A study was conducted to collect data from noninvasive photoplethysmography sensors to validate the CRM against an FDA-cleared CRI device. Participants were placed in a lower body negative pressure (LBNP) chamber and underwent exposure to a stepwise protocol of increasing negative pressure. Data from both algorithmic devices were evaluated using multiple approaches: median error, median absolute error, and pooled Pearson correlations. Data were compared from 20 participants through the duration of the experiment. Pooled correlation analysis between CRM and CRI for all subjects and LBNP steps achieved R^2^ = 0.859, showing strong linear agreement. Bland–Altman analysis revealed a near-zero mean bias between CRM and CRI (0.13%). Each metric allowed for a mean early measurement time of 20.52 min for CRM and 17.92 min for CRI. Consistent with our hypothesis, the CRM device performed similarly to CRI, demonstrating that both models can track compensatory status changes with progressive central hypovolemia. The early, real-time predictive tracking of compensatory changes can be valuable for continuous monitoring and triaging in prehospital trauma environments.

## 1. Introduction

Hemorrhage is the leading cause of preventable death in both civilian and military trauma [1,2]. Definitive hemorrhage control and fluid resuscitation are effective treatments, but early detection of the onset of shock is needed to properly triage casualty condition. A recent study determined that every minute of postponed detection—and thus delay in treatment—resulted in a 1.5% increase in 30-day mortality [3]. However, the onset of hemorrhagic shock and the associated physiological response is highly individualized, depending on compensatory mechanisms [4]. Compensatory physiological responses can mask the effects of blood loss, making traditional vital signs unreliable indicators of hemorrhagic shock [5], consequently resulting in delayed detection of hemorrhage and delivery of treatment [6]. Advanced monitoring technologies are needed that can accurately detect ongoing hemorrhage during early phases of compensation.

### 1.1. Compenstatory Reserve Devices

Machine learning approaches have been utilized for measuring compensatory status. These machine learning methodologies require high-fidelity physiological waveform data from hemorrhage to predict the state of hemodynamic decompensation [7]. In addition to high-fidelity physiological waveform capture, a repeatable model of central hypovolemia that induces hemodynamic decompensation was required to develop these algorithms. For this, the lower body negative pressure (LBNP) model simulates central hypovolemia by applying progressive, stepwise negative pressure, which redistributes the participant’s blood toward to the lower extremities [8]. This model has been extensively validated as a repeatable, noninvasive method of creating progressive hemorrhage [9].

The compensatory reserve index (CRI) was developed using machine learning methods utilizing more than 190 participant datasets that underwent LBNP protocols to identify the patient’s compensatory reserve by inducing central hypovolemia until a participant reached the point of hemodynamic decompensation [4]. The CRI algorithm continuously extracts features from noninvasive arterial signals and uses those features to track a patients’ compensatory status on a scale of 1 to 0 (full compensatory status to decompensation, respectively) [10]. CRI tracked patient compensatory status during conditions of central hypovolemia, allowing for earlier detection compared to traditional vital signs [11]. In addition, Nadler et al. demonstrated that CRI could also successfully track mild blood loss observed during blood donations [10]. The CRI algorithm has also been tested in subjects in a level I trauma center and it was demonstrated that the algorithm had an improved predictive capacity compared to systolic blood pressure [12]. A similar study showed that CRI had comparative predictive capability to arterial blood lactate during hemorrhage [13]. Overall, the CRI algorithm has been validated in both experimental and clinical settings as a potential indicator for ongoing hemorrhage shock.

A more recent approach developed by Techentin and co-workers used similar LBNP datasets to develop a deep learning neural network predictive model for compensatory status, termed the compensatory reserve measurement (CRM) [14]. Similarly, the CRM algorithm utilizes continuous noninvasive arterial waveform to quantify an individualized measure of physiological compensatory status during hypovolemia [15]. Studies have shown high correlations of CRM and percent blood loss in nonhuman primates [16,17]. Convertino et al. directly compared CRM to shock index, showing that CRM has improved tracking of reductions in central blood volume when compared to shock index [18]. Recent studies have demonstrated that CRM can be paired with noninvasive clinical and wearable sensors to provide accurate measurements utilizing a photoplethysmography (PPG) waveform [19,20,21].

While both algorithms have displayed exceptional predictive accuracy, translating both algorithms to clinical practice requires integration with wearable technologies. The CRI algorithm was incorporated into a wearable device developed by Flashback Technologies, Inc. and received Food and Drug Administration (FDA) De Novo Class II (DEN160020) acceptance in 2016, integrating CRI as the first methodology for providing real-time physiological compensatory status [22]. In addition, CRM has also been integrated with a portable clinical pulse oximetry technology for real-time implementation [20]. The CRM and CRI algorithms have been shown to provide equivalent values with retrospective analysis; however, their performance has not been compared using real-time measurements obtained from their respective hardware [23]. We hypothesized that values generated from the CRM algorithm would be statistically similar to those values generated from the CRI.

### 1.2. Scope of Work

To address the critical research gap of fielding devices suitable for early hemorrhage detection, here, we compare CRM and CRI models paired with their respective pulse oximeter hardware during progressive central hypovolemia in human subjects where each device provided compensatory reserve status in real-time. By doing so, we validated CRM against the FDA-cleared CRI device utilizing real-time measurements for the first time, confirming the functionality of each device for improving hemorrhage monitoring. The main contributions of this work are as follows:

• Direct comparison of two medical devices for measuring compensatory status using lower body negative pressure testing;
• Statistical analysis for a new CRM device against the FDA-cleared CRI device to characterize its similarities.

## 2. Materials and Methods

### 2.1. Lower Body Negative Pressure Research Protocol

#### 2.1.1. Ethical Approval and Study Population

The data capture and analysis in this study were conducted through Institutional Review Board (IRB)-approved human research protocols (H-24-013; H-26-011nh). A consent form and the study requirements were provided to potential participants to aid in gaining familiarity with the study. Inclusion criteria for enrollment of the study were (1) normotensive (<140/90) males or females, (2) between 18 and 65 years of age, (3) who were from military or civilian populations, (4) had a waist circumference between 22 and 42 inches, and (5) were willing to refrain from exercise and stimulants (including: caffeine, alcohol, chocolate, and herbal medications) for the 24 h prior to participation of the study.

#### 2.1.2. Experimental Setup

Upon being enrolled to participate in the study, the participants completed a health questionnaire and screening procedure with an Advanced Cardiovascular Life Support-certified medical monitor who verified compliance to withholding requirements 24 h prior to the study, exclusion criteria, and measurements for sensor usage for this study. Once the screening process had been completed and the participant was cleared to participate in the study, they were fitted with an appropriately sized kayak-style neoprene skirt and secured to the LBNP chamber.

**Figure 1:**
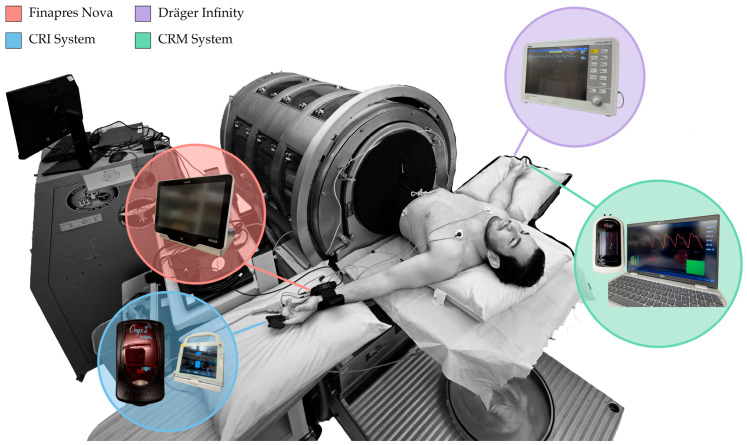
Experimental setup for the LBNP protocol and the sensor–device configurations. Four acquisition systems are highlighted with color coding (top left): continuous noninvasive finger arterial pressure and 3-lead ECG via the Finapres Nova (red); the CRI System (blue), comprising the Nonin Onyx II 9560 pulse oximeter paired with a Cybernet T10 tablet to generate CRI values; clinical vital-sign monitoring via the Dräger Infinity patient monitor (purple); and the CRM System (green), comprising the Masimo MightySat Rx pulse oximeter with custom USAISR software (V1.0) to generate CRM values.

Following the securement of the participant to the chamber, sensors were placed at their specified location. For this specific work, experimental sensors included 2 finger pulse oximeters (Nonin Onyx II 9560; Nonin Medical, Plymouth, MN, USA; Masimo MightySat Rx; Masimo, Irvine, CA, USA). A Masimo LNCS DCI pulse oximeter connected to a Dräger infinity patient monitor (Lübeck, Germany) and a 3-lead ECG connected to a Finapres Nova Noninvasive Blood Pressure system (Finapres Medical Systems, Enschede, Netherlands) were used for clinical monitoring of the participant. These sensors were used for data collection through the entire duration of the experiment. All the data streams of the sensors used in this study were temporally synchronized by recording both the device times and the master clock time at a common rollover event (e.g., HH:MM:00). Timestamp offsets were corrected post hoc to align all device data outputs to a common time reference for the study analysis. The complete experimental setup and sensor-device configuration are shown in Figure 1.

#### 2.1.3. LBNP Protocol

Once all sensors were placed on the participant, the standardized LBNP protocol was started. The protocol began with a 5 min baseline period at atmospheric pressure (0 mmHg), followed by a progressive stepwise reduction in chamber pressure. Negative pressure was increased in increments of −15 mmHg every 5 min until reaching −60 mmHg, after which pressure was incremented by −10 mmHg until the participant experienced presyncope symptoms (lightheadedness, nausea, tunnel vision, or a sudden drop in systolic blood pressure below 80 mmHg [24,25]) or completed the protocol at the end of the −100 mmHg step, whichever occurred first. After protocol completion or reaching presyncope, the chamber pressure was immediately released to atmospheric pressure, and participants were monitored during a 10 min recovery period.

### 2.2. Sensor-Device Configurations

For comparing CRM and CRI, two finger-based pulse oximetry devices were used to capture data during each LBNP experimental protocol. Each device acquired a PPG waveform and produced real-time compensatory reserve estimates on a common 0–1 (or 0–100%) scale, where 1 (100%) represents full compensatory status at baseline and 0 (0%) indicates imminent hemodynamic decompensation (HDD) [4].

These two devices each had respective device/software/algorithm pairings for the estimation of compensatory reserve. Compensatory reserve estimation algorithms were originally developed using arterial blood pressure waveforms collected via the Finometer Blood Pressure Monitor (TNO-TPD Biomedical Instrumentation, Amsterdam, The Netherlands) during controlled LBNP protocol [26]. The Nonin Onyx II 9560 pulse oximeter (Nonin Medical, Plymouth, MN, USA) was paired with a Cybernet T10 tablet (Cybernet Manufacturing, Irvine, CA, USA) to be able to generate CRI measurements [22]. The Masimo MightySat Rx (Masimo Corporation, Irvine, CA, USA) alongside a custom software solution developed at the U.S. Army Institute of Surgical Research (USAISR) provided CRM [20].

### 2.3. Compensatory Reserve Algorithms

Both CRI and CRM algorithms share the same mathematical basis with their training target being a calculated compensatory reserve value (Equation (1)).

(1) $$Compensatory\;Reserve=1-\frac{LBNP\left(t\right)}{{LBNP}_{HDD}}$$

where LBNP(t) is the negative pressure (mmHg) applied at time t and LBNP_HDD_ is the maximum negative pressure level tolerated by an individual during the research protocol resulting in the occurrence of HDD. This formulation assumes a linear relationship between applied LBNP and central blood volume loss and has been validated as a physiologically justified model for central hypovolemia [4,27]. Due to LBNP_HDD_ being determined individually by each participant’s own tolerance to progressive hypovolemia, compensatory reserve is subject-specific. It will range from 1.0 (100%) at baseline to 0.0 (0%) at the moment of HDD for each participant, irrespective of the absolute LBNP level reached.

In the absence of a direct physiological measure of compensatory reserve, this formulation represents the accepted reference training target related to the LBNP experimental framework and has been used consistently in prior studies [11,28,29,30].

#### 2.3.1. CRI

The underlying algorithm was developed by applying proprietary feature extraction and advanced machine learning methods to continuous noninvasive arterial blood pressure waveforms collected from 184 human LBNP subjects at the USAISR [3]. The CRI algorithm produces beat-to-beat estimates internally; CRI values were recorded from the device at 5 s intervals. The CRI estimation using the Nonin Onyx and its paired hardware has received FDA De Novo clearance [22].

#### 2.3.2. CRM

The underlying algorithm used for the estimation of CRM was based on a PPG waveform generated from the MightySat pulse oximeter using a one-dimensional convolutional neural network (1D CNN) trained on 5 s Finometer blood pressure waveforms from 194 human LBNP subjects [14]. The 20 subjects used in this study were independent of the original 194 subjects used to train CRM. However, our study did not exclude participants that were enrolled in previous LBNP studies, thus some subjects could have been part of the previous 194 subjects. The MightySat transmitted 31.25 Hz PPG waveform data to a custom Windows-based device for processing. Input waveforms were resampled at 100 Hz and normalized to a 0–1 amplitude scale using min–max normalization prior to inference. The algorithm generated CRM estimations at 1 Hz using a 5 s sliding window with a 1 s stride. These 1 Hz measurements are then fed into a 20 s averaging buffer to reduce beat-to-beat noise while maintaining the 1 Hz value updates [20]. The custom software solution and its pairing with the MightySat are investigational and have not received regulatory clearance.

Due to device implementation differences, in addition to the native CRM output at 1 Hz, CRM comparison was generated by decimating the 1 Hz CRM to 0.2 Hz by grabbing every fifth sample. This was done to provide a more direct temporal comparison to CRI, whose internal beat-to-beat estimates were recorded from the device at 5 s intervals.

### 2.4. Performance Metrics

The performance of CRM estimation was assessed relative to the FDA-cleared CRI. Prior to analysis, CRI outputs were normalized to a 0–100% scale so that all data streams were evaluated on a common scale consistent with CRM.

Agreement between devices was evaluated using multiple complementary approaches. Median Error (MdE) was used to determine the systematic bias of the CRM estimations relative to CRI, and Median Absolute Error (MdAE) assessed overall measurement accuracy independent of bias. Pooled Pearson correlation (R^2^) was computed across all paired time-matched observations to evaluate the linear association between CRM and CRI across the full compensatory reserve range. Bland–Altman analysis was performed to characterize the mean bias and 95% limits of agreement (LOA = bias ± 1.96 SD) between devices.

Trend-based early detection time was assessed for each modality using a sliding 5 min window, where a significant trend was defined as a slope drop of ≥15% compensatory reserve within the window. Detection time was defined as the first moment the trend criterion was met during the LBNP procedure compared to the experimental end time.

### 2.5. Statistical Analysis

For statistical analyses, three different tests were performed to highlight CRM and CRI comparisons using NCSS 2026 (Kaysville, UT, USA). First, correlations between CRM and CRI were assessed for statistical significance where the null hypothesis was the Pearson correlation being 0 between the two parameters. Second, the magnitudes of CRM and CRI on average for each participant were compared for equivalency. Data were assessed for normality by a Shapiro–Wilk test, followed by a paired *t*-test using two one-sided tests to assess equivalency in each direction. The null hypothesis was defined as CRM error relative to CRI is greater than 5%, comparable to the 5 bpm error margin set by ANSI standards [31,32]. Lastly, early prediction times for CRM were assessed for superiority relative to CRI prediction times. Data were assessed for normality by a Shapiro–Wilk test, followed by a paired *t*-test as defined by CRM early prediction times being longer to CRI prediction times. Throughout, precise *p*-values are provided in the accompanying text and presented in figures for ease of interpretation.

## 3. Results

### 3.1. Study Population

Datasets were collected from 20 participants through an IRB-approved protocol, as a sub-cohort of a larger prospective LBNP data collection effort. The participant population was recruited from the greater San Antonio, Texas, USA area. Demographic data are summarized in Table 1. All twenty participants had data recorded from the compensatory reserve devices through the entire duration of the experiment. The median completed LBNP step was 70 mmHg.

### 3.2. Comparitive Analysis

**Figure 2:**
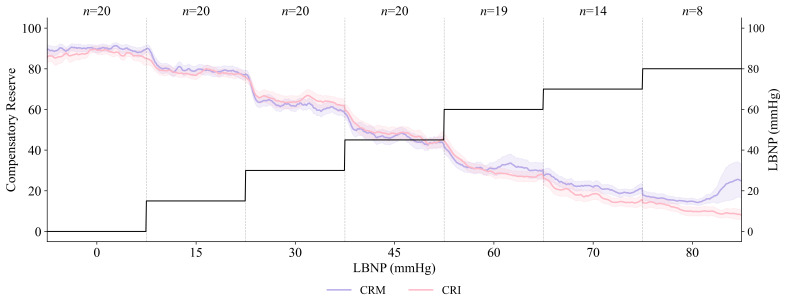
Comparison of compensatory reserve monitoring devices across simulated hemorrhage using lower body negative pressure. Group mean ± SEM compensatory reserve across LBNP steps for CRM and CRI compared to the lower body negative pressure protocol. LBNP magnitudes are displayed as percent values.

Average results were compiled across the LBNP step profile for the CRM and CRI real-time compensatory reserve estimations (Figure 2). Pooled MdE was 0.0255 [IQR: −5.21, 5.00], and pooled MdAE was 5.09 [IQR: 2.13, 9.89], indicating minimal systematic bias between devices. Subjects who reached presyncope prior to a given LBNP were excluded from subsequent steps, resulting in decreasing sample sizes at higher levels.

**Figure 3:**
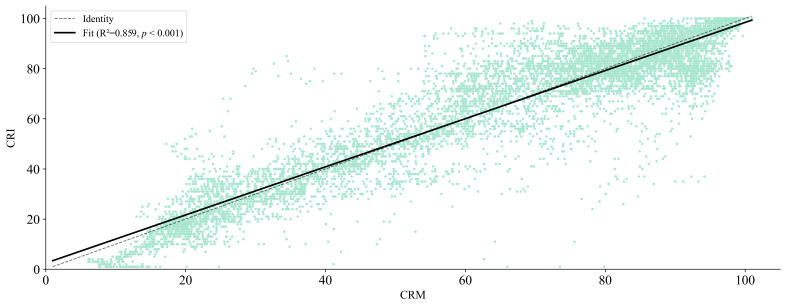
Pooled scatter plot of compensatory reserve measurements from CRM and CRI across all LBNP steps and subjects (*n* = 20, 8599 paired observations). The dashed line represents the line of identity. The solid line represents the linear regression fit (R^2^ = 0.858).

Pooled correlation analysis between CRM and CRI compensatory reserve values across all subjects and LBNP steps revealed strong linear agreement (R^2^ = 0.859, *p* < 0.0001; *n* = 8599 paired observations, Figure 3). The linear regression fit closely approximated the line of identity, suggesting minimal proportional bias between devices across the full compensatory reserve range. The regression *p*-value of less than 0.0001 indicates very strong evidence that the regression coefficient between CRM and CRI is not zero.

**Figure 4:**
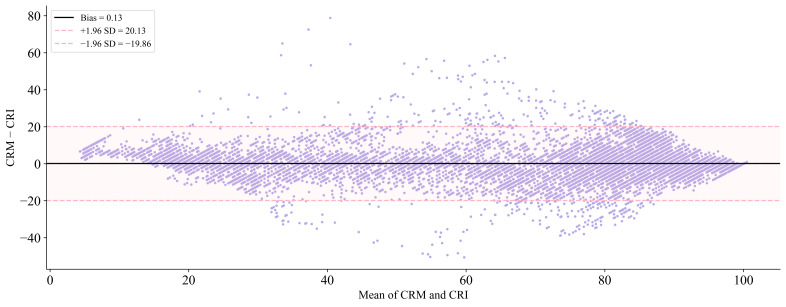
Bland–Altman analysis of agreement between CRM and CRI compensatory reserve measurements. The solid line indicates the mean bias (0.13) and the dashed lines indicate the 95% limits of agreement (−19.85 to 20.13). Data are pooled across 20 subjects and 8599 paired observations during progressive LBNP-simulated hemorrhage.

Bland–Altman analysis revealed a near-zero mean bias between CRM and CRI (0.13%), indicating no systematic over- or under-estimation by CRM relative to the FDA-cleared CRI (Figure 4). The 95% limits of agreement spanned -19.86 to 20.13 (SD = 10.20), representing a total agreement range of approximately 40% across the full compensatory reserve scale. Comparing subject-level prediction mean values for CRM (64.57%, *n* = 20 subjects) and CRI (64.20%, *n* = 20 subjects), results for CRM were highly equivalent to CRI within a 5% acceptable error band with a *p*-value of 0.00021.

**Figure 5:**
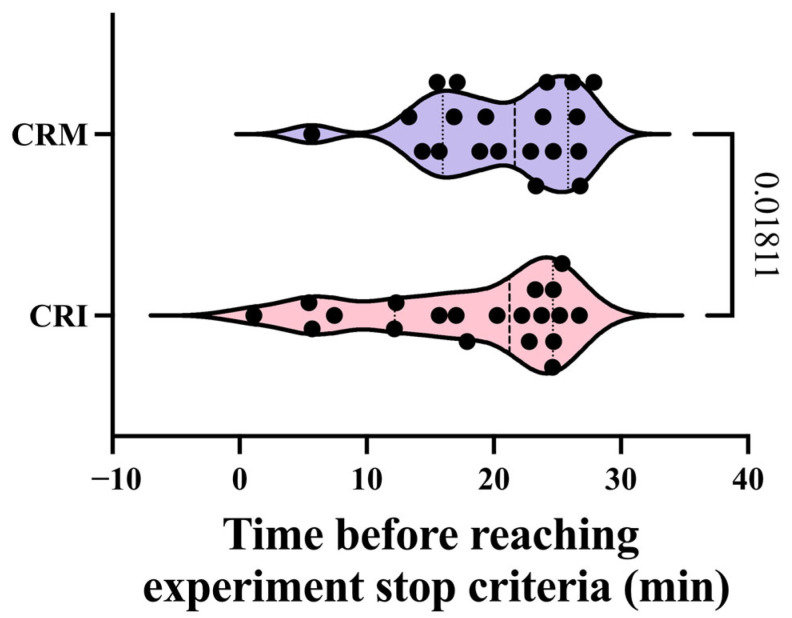
Lead time (min) before experimental stop criteria for CRM and CRI (*n* = 20). Statistics for detection times of CRM relative to CRI were performed by paired *t*-test.

Ultimately, CR status can improve detection of the onset of decompensation, so early prediction capability for the onset of decompensated shock was compared for CRM and CRI based on declination in CR value over time (Figure 5). CRM and CRI allowed for a mean early detection time of 20.52 ± 5.82 min for CRM and 17.92 ± 7.95 min for CRI. We assessed if CRM detection times were superior to CRI detection times using a paired *t*-test. Using an inferiority limit of 0%, *p*-value for the comparison was 0.01811, indicating CRM was strongly superior to CRI for detection time.

## 4. Discussion

Hemorrhage continues to be the leading cause of preventable death in both civilian and combat casualty care [1,33]. There is extensive research on treatments for hemorrhage control and fluid resuscitation; however, early indicators for the onset of hemorrhagic shock are needed to accurately assess and triage casualties [34]. Current symptoms that accompany severe blood loss such as clammy skin, weak pulse and vital sign instability are inconsistent and often are late indicators since they can be masked by physiological compensatory mechanisms [35]. Machine learning methodologies have been used to create metrics and devices that allow real-time monitoring of physiological compensatory status. The CRI and CRM metrics utilize LBNP datasets and advanced algorithms to allow early estimates of compensatory status and were later incorporated with portable wearable hardware for real-time measurements. The present study builds on previous work by comparing CRI and CRM algorithms in a human model of central hypovolemia utilizing their respective hardware to collect real-time measurements. This approach provided for the first real-time validation of the CRM model against an FDA-cleared device and evaluates each device’s functionality for early and accurate compensatory status during progressive reductions in central blood volume.

Our findings suggest that the CRM maintains a strong linear correlation (R^2^ = 0.859) with CRI. Comparison metrics showed minimal systematic bias between CRI and CRM. CRM had notably higher MdAE (5.09) than MdE (0.0255), indicating some error between both models. Figure 1 shows some deviation between CRM and CRI at lower pressure steps (−60 mmHg and below) in the LBNP protocol, which likely stems from two factors. First, distinguishable features in physiological signals degrade as humans approach the clinical condition of presyncope, reducing estimation accuracy. Second, reduced sample size due to individual variability in participant intolerance to central hypovolemia is associated with increased SEM, potentially magnifying minor model differences in performance.

The Bland–Altman Analysis further corroborated similarities between CRM and CRI. While there was no systematic over- or underestimation by CRM relative to CRI, we observed a similar measurement deviation as pressure steps increased. Crucially, both metrics provided a significant early warning window, with mean detection times exceeding 17 min. While the CRI showed earlier mean time of measurement compared to CRM, the lack of statistical non inferiority between CRM and CRI suggests that both metrics offer comparable utility in time-critical scenarios. These mean early estimations have important utility for triaging casualties, with both metrics allowing for a significant time window to perform time-critical interventions during casualty scenarios [36].

Although the Bland–Altman analysis demonstrated near zero mean bias, the 95% LOA (−19.86% to +20.13%) indicate that the instantaneous CRM and CRI values can differ by up to approximately 20 percentage points in an individual. The two devices should not be regarded as interchangeable for isolated, single-timepoint readings, and a lone value should not by itself drive a triage decision. Compensatory reserve, however, is informative as a continuously trending signal: the clinically actionable information lies in the direction and rate of decline toward decompensation rather than in the absolute magnitude at any single instant. Our results support this on two different fronts. Firstly, at the subject level, the two devices were statistically equivalent in mean compensatory reserve (64.57% vs. 64.20%), indicating that moment-to-moment variability largely averages out and reflects noise around a shared central estimate rather than systematic divergence. Secondly, our early-detection analysis was itself trend-based, defining onset by a sustained decline over a sliding window rather than by any absolute threshold. Under this analysis, CRM was non-inferior to CRI, supporting that the LOAs do not translate into divergent trend behavior. Taken together, these findings indicate that CRM, like the FDA-cleared CRI, is best deployed as a trend-monitoring tool, and that interpreting values as trends or aggregating them over time mitigates the point-wise variability indicated by the LOA.

There are limitations worth discussing. Firstly, the proprietary nature of the CRI algorithm fails to provide transparency of how the algorithm computes and calculates the compensatory status of the patient. While accurate, the CRI model could have some bias that would be difficult to identify due to the “black box” nature of the machine learning model. Secondly, CRM is still an investigational device and has not reached FDA approval pathways. While the results of this study demonstrate statistical equivalence to the FDA-cleared CRI algorithm, further work is needed to fully assess the safety and effectiveness of CRM in different scenarios of reduced circulating blood volume. Thirdly, this study had a population of healthy individuals undergoing a controlled hemorrhage simulation. This allowed for a more controlled comparison between CRM and CRI models but lacks the variability observed in real world scenarios. In addition, due to the controlled laboratory setting of this study and previous studies used to develop the CRM algorithm, the data collected contained minimal motion artifacts or other forms of signal noise. This is unrepresentative of real-world scenarios where various forms of signal noise can be introduced, such as during en route care. The CRM CNN model is currently unequipped with the means to identify and properly handle this kind of data. Future work would need to characterize this data and develop methodologies to process the waveform before being used for CRM inference. However, direct comparisons of accuracy, sensitivity and specificity of CRM across clinical conditions of hemorrhage and trauma provide compelling evidence that the CRM has been translated to various clinical trauma-relevant environments [17].

## 5. Conclusions

For the first time, the CRM algorithm used for the real-time assessment of compensatory status was validated against the FDA-cleared CRI algorithm in a controlled simulated hemorrhage scenario. Consistent with our hypothesis, both metrics proved to track hemodynamic changes in identical conditions of ongoing progressive central hypovolemia designed to simulate progressive ongoing hemorrhage. The comparison metrics showed minimal systemic bias between technologies designed to measure the real-time values of compensatory reserve. Both models demonstrated early, real-time predictive tracking of compensatory changes, proving to be potential advancements for continuous monitoring and triage in prehospital and casualty trauma. Future work will evaluate these devices in clinical and military settings to further assess their utility in real-world environments.

## Figures and Tables

**Table 1:** Participant demographics and baseline values. Data presented as mean ± SD, *n* (%), or median [IQR].

Characteristic	Value
Participants, *n*	20
Age, years	33.2 ± 10.6
Sex, M/F	13 (65%)/7 (35%)
*Race*	
White	16 (80%)
Non-white	4 (20%)
*Ethnicity*	
Hispanic	3 (15%)
Non-Hispanic	14 (70%)
Not reported	3 (15%)
*Additional Info*	
BMI, kg/m^2^	26.4 ± 5.2
Resting SBP, mmHg	116 ± 13
Resting DBP, mmHg	76 ± 9
Resting HR, bpm	71 ± 14
Final LBNP Step Reached, mmHg	70 [60–80]

## Data Availability

The data presented in this study are not publicly available because they have been collected and maintained in a government-controlled database located at the U.S. Army Institute of Surgical Research. This data can be made available through the development of a Cooperative Research and Development Agreement (CRADA) with the corresponding author.

## Abbreviations

The following abbreviations are used in this manuscript:

CRADA	Collaborative research and development agreement
CRM	Compensatory reserve measurement
CRI	Compensatory reserve index
FDA	Food and drug administration
HDD	Hemodynamic decompensation
IRB	Institutional review board
LBNP	Lower body negative pressure
LOA	Limits of agreement
MdAE	Median absolute error
MdE	Median error
PPG	Photoplethysmography
